# Evaluation de la capacité opérationnelle des établissements sanitaires de première ligne dans la prise en charge du paludisme en Côte d’Ivoire

**DOI:** 10.11604/pamj.2019.34.16.19932

**Published:** 2019-09-06

**Authors:** David Guanga Meless, Annita Emeline Hounsa, Abou Dramane Sangaré, Adama Sanogo Pongathié, Julie Sackou Kouakou, Mamadou Samba, Luc Kouadio

**Affiliations:** 1 Département de Santé Publique, UFR Odontostomatologie, Université Félix Houphouët-Boigny, Abidjan, Côte d’Ivoire; 2 Département de Santé Publique, Hydrologie et Toxicologie, UFR des Sciences Pharmaceutiques et Biologiques, Université Félix Houphouët-Boigny, Abidjan, Côte d’Ivoire; 3 Direction de l’Information et de l’Informatique Sanitaire, Ministère de la Santé et de l’Hygiène Publique, Abidjan, Côte d’Ivoire

**Keywords:** Côte d’ivoire, capacité opérationnelle, établissements de santé de première ligne, paludisme, Ivory Coast, operational capacity, first line health facilities, malaria

## Abstract

**Introduction:**

Les établissements de santé (ES) possèdent-ils les ressources de base pour la prise en charge des cas de paludisme? L’objectif de notre étude était d’analyser la Capacité Opérationnelle (CO) des ES de première ligne de la Côte d’Ivoire dans la prise en charge du paludisme.

**Méthodes:**

La méthodologie SARA a été utilisée pour réaliser une étude transversale descriptive du 10 au 30 juillet 2016. La CO pour la prise en charge du paludisme a exprimé la disponibilité moyenne de 9 éléments traceurs répartis en 3 domaines: (i) personnel et directives; (ii) capacité de diagnostic; (iii) médicaments et produits. Cette CO a été évaluée par le calcul d’un indice puis comparé entre les ES selon l’instance gestionnaire et la zone géographique à l’aide du test du Chi2 pour un risque de première espèce α fixé à 0,05.

**Résultats:**

Parmi les 818 ES, 651(79,6%) étaient du secteur public et 487(59,5%) situés en zone rurale. La CO des ES de première ligne était de 74,5%. Cette CO était plus élevée dans le public (81,3%) que dans le privé (48,8%) (p < 10^-3^) ainsi qu’en zone rurale (82,7%) par rapport à la zone urbaine (62,9%) (p < 10^-3^).

**Conclusion:**

Les ES de première ligne de la Côte d’Ivoire disposaient en 2016 des ressources de base pour une prise en charge des cas de paludisme. Il est nécessaire de mettre l’accent sur le renforcement du système de santé dans son ensemble en plus de la prévention.

## Abstract (en)

**Introduction:**

Do health facilities (HF) have basic resources needed to manage malaria? The purpose of our study was to analyze the operational capacity (OC) of first-line health facilities in Ivory Coast in the management of malaria.

**Methods:**

SARA methodology was used to conduct a descriptive cross-sectional study from 10 to 30 July 2016. The operational capacity in the management showed an average availability of 9 identification tracers divided in 3 areas: (i) staff and guidelines; (ii) capacity of diagnosis; (iii) drugs and products. This operational capacity was assessed through the calculation of an index and then compared with the health facilities according to the management authority and the geographical area using Chi-square test with p-values α fixed at 0.05.

**Results:**

Out of 818 HFs, 651(79.6%) were in the public sector and 487(59.5%) were located in the rural area. The operational capacity of first line health facilities was 74.5%. This OC was higher in the public sector (81.3%) than in the private sector (48.8%) (p < 10^-3^) as well as in the rural area (82.7%) compared to the urban area (62.9%) (p < 10^-3^).

**Conclusion:**

In 2016, first line health facilities in Ivory Coast had basic resources needed to manage malaria. It is necessary to focus on the need to strengthen health facility services in addition to prevention.

## Introduction

Le paludisme demeure de par sa fréquence et son ampleur, la première endémie tropicale. Bien qu’il ait été enregistré de 2000 à 2017 une baisse de son incidence et de sa mortalité au niveau mondial, l’Afrique subsaharienne compte pour une part importante et disproportionnée de la charge mondiale du paludisme. En 2017, l’on recensait 92% des cas de paludisme et 93% des décès dus à la maladie dans cette région [1]. En Côte d’Ivoire, le paludisme constitue le premier motif de consultation et d’hospitalisation au niveau des établissements de santé (ES) avec une incidence de 154,58‰ en 2016 [2]. Avec un nombre de décès estimé à 3222 , la Côte d’Ivoire fait partie des 18 pays où se sont concentrés en 2017, 80% des décès dus au paludisme dans le monde [1]. Devant la mortalité élevée due au paludisme en Côte d’Ivoire, l’on pourrait se demander si les ES possèdent les ressources de base nécessaires pour mettre en œuvre la stratégie de lutte à travers les mesures préventives comme l’utilisation des moustiquaires imprégnées d’insecticides et les mesures curatives se basant sur le diagnostic rapide et le traitement correct des cas cliniques? En résumé, ces ES disposent-ils d’une capacité opérationnelle? Le principe de base de la « Capacité Opérationnelle » (CO) [3] repose sur le cadre traditionnel de la qualité des soins comprenant 3 dimensions: la structure (caractéristiques physiques et organisationnelles), le processus (soins fournis) et les résultats (effets des soins sur la santé des patients) [4-6]. La structure est importante car constitue le prérequis à la qualité des soins et influence le processus qui à son tour affecte les résultats de santé [5]. La méthodologie SARA (*The Service Availability and Readiness Assessment*), une des enquêtes sur les ES le plus couramment utilisée, vise à mesurer la CO en utilisant un ensemble d’éléments traceurs pour déterminer dans quelle mesure un établissement aussi bien public que privé remplit ou non les conditions pour permettre la prestation des services de santé [7]. Il est donc important que les systèmes de santé soient capables d’évaluer et de suivre la CO afin de réagir de manière appropriée aux besoins des ES notamment ceux de première ligne qui constituent la porte d’entrée du système de santé. La signalisation des études publiées jusqu’en décembre 2018 sur le paludisme dans les bases de données et moteurs de recherche comme PubMed/Medline, Google scholar avec les mots clés « Malaria », « Côte d’Ivoire » ont montré qu’aucune ne s’est intéressée à la CO des ES dans la prise en charge du paludisme. Ainsi l’objectif de notre étude était d’évaluer la capacité opérationnelle des établissements sanitaires de première ligne de la Côte d’Ivoire dans la prise en charge du paludisme dans le but de contribuer au contrôle et à l’élimination de cette pathologie dans ce pays.

## Méthodes

**Echantillonnage:** l’étude SARA est une enquête nationale, de type transversal à visée descriptive qui a concerné tous les ES aussi bien publics que privés, dans les 20 régions sanitaires de la Côte d’Ivoire. Toutes les structures sanitaires gérées par le ministère en charge de la santé ont été considérées comme ES publics. Les ES privés à but lucratif et confessionnelles ont été regroupées dans la catégorie des structures privées. La base de sondage utilisée était la liste des ES du Ministère de la Santé et de la Lutte Contre le Sida (MSLS) de l’année 2014. En Côte d’Ivoire, l’offre des soins est organisée en 3 niveaux. Le Niveau 1 qui constitue la porte d’entrée du système de santé est constitué des ES de première ligne (ou de premier contact). Le Niveau 2 est constitué des Centres Hospitaliers Régionaux (CHR), des Hôpitaux Généraux (HG) et des cliniques privées. Le Niveau 3 est formé des Centres Hospitaliers Universitaires (CHU) et des polycliniques. Le nombre d’ES a été calculé pour l’ensemble des ES des 3 niveaux, à partir de la formule de Schwartz, avec une fréquence estimative des ES qui devraient avoir les éléments traceurs recherchés par l’enquête SARA de 50%, une précision de 10% et un risque d’erreur alpha à 5%. La taille brute de l’échantillon des ES de première ligne à inclure était de 777 structures. Cet effectif a été majoré de 10%, soit 855 ES à sélectionner, proportionnellement au nombre d’ES par instance gestionnaire, par zone géographique et par région.

**Collecte des données:** la collecte des données, réalisée du 10 au 30 juillet 2016, s’est faite à l’aide du questionnaire standard SARA, développé par l’OMS et adapté au contexte du pays en prenant en compte les types d’établissement et leurs instances gestionnaires; les lignes directrices nationales sur les services et la politique nationale des médicaments et intrants [7]. Neuf éléments traceurs présents au niveau des ES de première ligne offrant des services de prise en charge du paludisme ont été collectés. Ces éléments traceurs étaient répartis en 3 domaines. Le premier domaine « Personnel et directives » comportait 4 éléments traceurs: (1) directives sur le diagnostic et le traitement du paludisme; (2) directives sur le Traitement Préventif Intermittent (TPI); (3) un membre du personnel au moins formé sur le diagnostic et le traitement du paludisme dans les 3 dernières années; (4) un membre du personnel au moins formé sur le TPI dans les 3 dernières années. Le deuxième domaine « capacité de diagnostic » comportait 1 élément traceur: (5) capacité de diagnostic du paludisme. Il a pris en compte le Test de Diagnostic Rapide (TDR) ou l’examen de la goutte épaisse/frottis sanguin avec le matériel, les réactifs nécessaires (microscope, lames) et la présence de microscopiste certifié. Le troisième domaine « médicaments et produits » comportait 4 éléments traceurs: (6) paracétamol; (7) médicament pour le TPI adressé aux femmes enceintes; (8) deux antipaludiques de première ligne au moins en stock; (9) moustiquaires imprégnées d’insecticide à longue durée d’action (MILDA) ou coupons disponibles pour la distribution. Les 2 antipaludiques de première ligne ont concerné les combinaisons thérapeutiques à base d’artémisinine (CTA) et l’association artésunate+amodiaquine. Le médicament pour le TPI était l’association sulfadoxine+pyriméthamine. Pour analyser la disponibilité réelle en antipaludiques et en test de diagnostic, les ruptures de stocks des TDR et des combinaisons à base d’artémisinine au cours des 4 dernières semaines précédant l’enquête ont été recherchées. Le questionnaire a été préalablement testé dans des ES non sélectionnés pour l’enquête SARA en Côte d’Ivoire. Les données ont été recueillies par un binôme d’enquêteurs contrôlés par des superviseurs et coordonnateurs, tous formés pour la collecte des données de l’enquête SARA. Dans chaque ES, en plus de l’entretien en face à face avec les interlocuteurs indiqués pour fournir les informations recherchées, l’observation a permis aux enquêteurs de vérifier la disponibilité, puis le fonctionnement des équipements ainsi que la date de péremption des médicaments et consommables le jour de la visite. Des documents tels que les registres, rapports d’activités et fiche de stock ont été passés en revue.

**Analyse des données:** les données collectées sur support papier ont été saisies et analysées avec le logiciel CS Pro. A partir de la base de données constituée, la matrice SARA_chartbook_v2.2 mis à disposition par l’OMS, a été renseignée pour générer les résultats de l’enquête SARA. Un score moyen de disponibilité des éléments traceurs qui correspond au pourcentage d’ES disposant de l’élément a été calculé. La CO pour la prise en charge du paludisme, exprimé en pourcentage, a été évaluée par le calcul d’un indice de CO qui correspond à la moyenne des scores moyens de disponibilité des 9 éléments traceurs. Le test du Chi2 pour échantillons indépendants a été utilisé pour comparer les CO selon l’instance gestionnaire (public/privé) et la zone géographique (rurale/urbaine) avec un risque de première espèce α fixé 0,05.

## Résultats

Sur les 855 ES sélectionnés, 818 ont été enquêtées, soit un taux de réponse de 95,7%. Les ES non répondant correspondaient à des structures sélectionnées mais non fonctionnelles au moment de l’enquête.

**Description des ES de première ligne selon l’instance gestionnaire, la zone géographique et les ruptures de stock en TDR et CTA:** parmi les 818 ES inclus dans l’étude, 651(79,6%) étaient du secteur public et 487(59,5%) étaient situés en zone rurale. L’évaluation de la disponibilité des tests de diagnostic et des médicaments a montré que les fréquences des ES ayant connu au moins une rupture en stock durant les 4 dernières semaines précédant l’enquête étaient de 23,7% pour les tests de diagnostic rapide du palusdisme et de 13,9% pour les combinaisons à base d’Artémisinine (Tableau 1).

**Tableau 1:** Répartition des ES de première ligne selon l’instance gestionnaire, la zone géographique et la présence ou non de rupture de stock en CTA et TDR

	Effectif (n)	Pourcentage (%)
**Instance gestionnaire**		
Public	651	79,6
Privé	167	20,4
**Zone géographique**		
Urbaine	331	40,5
Rurale	487	59,5
**Rupture de stock en TDR**		
Oui	194	23,7
Non	624	76,3
**Rupture de stock en CTA**		
Oui	114	13,9
Non	704	86,1

ES : Etablissements de Santé; TDR: Test de Diagnostic Rapide; CTA: Combinaison à base d’Artémisinine

**Disponibilité des 9 éléments traceurs de CO pour la prise en charge du paludisme et rupture en stock des TDR et CTA dans les ES de première ligne:** moins du quart des ES (23,5%) disposaient de l’ensemble des 9 élements traceurs de CO pour la prise en charge du paludisme. Le score de disponibilité des éléments traceurs du domaine 1 relatif aux “personnel et directives” était de 67,5%. Environ 4 ES sur 5 disposaient de directives et de personnel formé au diagnostic et traitement du paludisme, plus de 60% des ES avaient des directives sur le TPI. Neuf ES sur dix avaient la capacité de faire le diagnostic du paludisme. Le score moyen de disponibilité des éléments traceurs du domaine 3 relatif aux “médicaments et produits” était de 78,5%, ce qui correspondait à près de 4 ES sur 5 disposant de médicaments et produits de base pour la prise en charge du paludisme. Le score de disponibilité le plus élevé a été retrouvé au niveau des antipaludiques de première ligne avec 85,0% des ES qui en disposaient. En moyenne les ES avaient 7 éléments traceurs disponibles sur les 9, ce qui correspondait à un indice de CO de 74,5% (Tableau 2).

**Tableau 2:** Description du score moyen de disponibilité des éléments traceurs pour la prise en charge du paludisme par domaines et par éléments traceurs et indice de CO des ES de première ligne

Élements traceurs (ET) par domaines (D) et rupture de stock	ES ayant les éléments traceurs	
	Oui n (%)	Non n (%)
**D_1_: Personnel et Directives**		
ET_1_: Directives sur le diagnostic et traitement	630 (77,0)	188 (23,0)
ET_2_: Directives sur le TPI	504 (61,6)	314 (38,4)
ET_3_: Agent formé au diagnostic et traitement	623 (76,2)	195 (23,8)
ET_4_: Agent formé sur le TPI	452 (55,3)	366 (44,7)
***Les 4 éléments traceurs du domaine 1***	**552 (67,5)**	**266 (32,5)**
**D_2_: Capacité de diagnostic**		
ET_5_: Capacité de diagnostic du paludisme	720 (88,0)	98 (12,0)
**D_3_: Médicaments et produits**		
ET_6_: Paracétamol comprimé	646 (79,0)	172 (21,0)
ET_7_: Médicaments pour le TPI	606 (74,1)	212 (25,9)
ET_8_: Antipaludiques de première ligne	694 (85,0)	124 (15,0)
ET_9_: MILDA	624 (76,3)	194 (23,7)
***Les 4 éléments traceurs du domaine 3***	**642 (78,5)**	**176 (21,5)**
**Tous les 9 éléments traceurs**	**192 (23,5)**	**626 (76,5)**
**Indice de CO: 74,7%**		

CO: Capacité Opérationnelle; ES: établissements de santé; ET: élément traceur, TPI: traitement préventif intermittent, MILDA: moustiquaires imprégnées d’insecticide à longue durée d’action

**CO pour la prise en charge du paludisme et rupture en stock de TDR et CTA des ES de première ligne selon l’instance gestionnaire et la zone géographique:** le score de disponibilité de l’ensemble des 9 éléments traceurs au niveau des ES de première ligne selon l’instance gestionnaire et la zone géographique était reparti comme suit: 27,3% pour le secteur public contre 8,4% pour le secteur privé et 16,6% pour la zone urbaine contre 28,1% pour la zone rurale (Tableau 3, Tableau 4). La comparaison selon l’instance gestionnaire a montré que les scores de disponibilité des éléments traceurs étaient significativement plus élevés dans le secteur public que le secteur privé quel que soit l’élément traceur considéré. L’indice de CO pour la prise en charge du paludisme était plus élevé dans le secteur public 81,3% que dans le secteur privé (48,8%) (p < 10^-3^). Il n’y avait pas de différence de rupture de stock selon l’instance gestionnaire, aussi bien pour les TDR du paludisme que pour les CTA (Tableau 3). L’analyse selon la zone géographique a montré que les scores de disponibilité des éléments traceurs étaient plus élevés en zone rurale qu’en zone urbaine pour tous les éléments traceurs sauf pour la disponibilité du “paracétamol comprimé” qui était de 76,1% en zone urbaine et 80,9% en zone rurale (p=0,100). L’indice de CO pour la prise en charge du paludisme était plus élevé en zone rurale (82,7%) qu’en zone urbaine (62,9%) (p < 10-3). Il n’y avait pas de différence de rupture de stock selon la zone géographique, aussi bien pour les TDR du paludisme que pour les CTA (Tableau 4).

**Tableau 3:** Description du score moyen de disponibilité des éléments traceurs pour la prise en charge du paludisme par domaines, par éléments traceurs, et indice de CO des ES de première ligne, selon l’instance gestionnaire

Élements traceurs (ET) par domaines (D) et rupture de stock	Instance gestionnaire		P value
	Public (n=651)n (%)	Privé (n=167)n (%)	
**D_1_: Personnel et Directives**			
ET_1_: Directives sur le diagnostic et traitement	544 (83,6)	86 (51,5)	**< 0,001**
ET_2_: Directives sur le TPI	463 (71,0)	41 (24,6)	**< 0,001**
ET_3_: Agent formé au diagnostic et traitement	538 (82,6)	85 (50,9)	**< 0,001**
ET_4_: Agent formé sur le TPI	401 (61,6)	51 (30,5)	**< 0,001**
**D_2_: Capacité de diagnostic**			
ET_5_: Capacité de diagnostic du paludisme	602 (92,5)	118 (70,7)	**0,011**
**D_3_: Médicaments et produits**			
ET_6_: Paracétamol comprimé	526 (80,8)	120 (71,9)	**< 0,001**
ET_7_: Médicaments pour le TPI	536 (82,3)	70 (41,9)	**< 0,001**
ET_8_: Antipaludiques de première ligne	597 (91,7)	97 (58,1)	**< 0,001**
ET_9_: MILDA	558 (85,7)	66 (39,5)	**< 0,001**
**Ruptures en Test de Diagnostic Rapide (TDR)**	147 (22,6)	47 (28,1)	0,131
**Ruptures en Combinaisons Thérapeutiques à base d’Artémisinine (CTA)**	95 (14,6)	19 (11,4)	0,154
**Indice de CO,*% [IC_95%_]***	**81,3 [79,6-82,4]**	**48,8 [44,3-53,8]**	**< 0,001**

CO: capacité opérationnelle; ET: éléments traceur, TPI: traitement préventif intermittent, MILDA: moustiquaires imprégnées d’insecticide à longue durée d’action

**Tableau 4:** Description du score moyen de disponibilité des éléments traceurs pour la prise en charge du paludisme par domaines, par éléments traceurs, et indice de capacité opératoire des ES de première ligne, selon la zone géographique

Élements traceurs (ET) par domaines (D)	Zone géographique		P value
	Urbaine (n=331)n (%)	Rurale (n=487)n (%)	
**D_1_: Personnel et Directives**			
ET_1_: Directives sur le diagnostic et traitement	216 (65,3)	414 (85,0)	**< 0,001**
ET_2_: Directives sur le TPI	146 (44,1)	358 (73,5)	**< 0,001**
ET_3_: Agent formé au diagnostic et traitement	214 (64,7)	409 (84,0)	**< 0,001**
ET_4_: Agent formé sur le TPI	142 (42,9)	310 (63,7)	**< 0,001**
**D2: Capacité de diagnostic**			
ET_5_: Capacité de diagnostic du paludisme	271 (81,9)	449 (92,2)	**< 0,001**
**D_3_: Médicaments et produits**			
ET_6_: Paracétamol comprimé	252 (76,1)	394 (80,9)	0,100
ET_7_: Médicaments pour le TPI	191 (57,7)	415 (85,2)	**< 0,001**
ET_8_: Antipaludiques de première ligne	246 (74,3)	448 (92,0)	**< 0,001**
ET_9_: MILDA	195 (58,9)	429 (88,1)	**< 0,001**
**Ruptures en Test de Diagnostic Rapide (TDR)**	87 (26,3)	107 (22,0)	0,284
**Ruptures en Combinaisons Thérapeutiques à base d’Artémisinine (CTA)**	48 (14,5)	66 (13,6)	0,700
**Indice de CO, *% [IC_95%_]***	**62,9 [59,7-66,3]**	**82,7 [81,5-84,5]**	**< 0,001**

ES: établissements de santé; ET: éléments traceur; TPI: traitement préventif intermittent; MILDA: moustiquaires imprégnées d’insecticide à longue durée d’action

## Discussion

Cette étude avait pour objectif d’évaluer la CO des ES de première ligne de la Côte d’Ivoire dans la prise en charge du paludisme à partir de la méthodologie SARA [7]. Une des limites relevées dans cette étude est inhérente à la méthodologie SARA qui ne permet pas d’évaluer le processus c’est à dire l’utilisation des ressources permettant une bonne prise en charge des cas de paludisme [3]. Néanmoins, notre étude fournit des données essentielles sur la CO des ES de première ligne de la Côte d’Ivoire. C’est la première à présenter cette CO pour la prise en charge du paludisme au niveau des ES de première ligne selon l’instance gestionnaire et la zone géographique. Le facteur déterminant dans la prise en charge efficace du paludisme au niveau des ES est la disponibilité des ressources de base concernant le diagnostic et traitement de cette pathologie. La comparaison des ruptures de stock en TDR et CTA n’a pas montré de différence significative ni entre les secteurs public et privé, ni entre zone rurale et urbaine. La prise en compte de ces ruptures de stock dans l’analyse des données permet d’éliminer l’éventuelle influence des ruptures de stock dans la comparaison des CO selon l’instance gestionnaire et selon la zone rurale. Dans cette étude, la CO des ES de première ligne de la Côte d’Ivoire a été estimée à 74,9%. Les rapports disponibles de certains pays de l’Afrique subsaharienne ayant réalisés l’enquête SARA ont présenté la CO pour la prise en charge du paludisme au niveau de tous les ES et pas spécifiquement au niveau des ES de première ligne. Cette CO pour la prise en charge du paludisme au Niger était de 68% en 2015 [8], au Bénin de 69% en 2015 [9] et en Mauritanie de 46% en 2016 [10].

Nos résultats ont montré que les ES de première ligne du secteur public et ceux situés en zone rurale étaient plus équipés que ceux du secteur privé et ceux situés en zone urbaine. La prévalence du paludisme étant plus fréquente en zone rurale qu’en zone urbaine [11], la plus grande disponibilité des ressources de base dans cette zone semble justifiée. Cette bonne disponibilité des ressources de base pour la prise en charge du paludisme au niveau des ES de première ligne du secteur public et en zone rurale contraste avec la disponibilité des ressources de base pour la prestation des soins de santé généraux. En effet, en 2016 au niveau des ES de première ligne du secteur public et en zone rurale l’on manquait d’électricité, d’eau courante, de réactifs de laboratoire, de moyens matériels et logistiques, de médicaments etc [12]. Notre étude a montré que l’on ne manque pas de tests de diagnostic du paludisme, ni de personnel formé au diagnostic et traitement du paludisme, ni de médicaments antipaludiques, ni de directives pour la prise en charge du paludisme. Le paludisme bénéficie du financement du Fonds Mondial de Lutte contre le Sida, la Tuberculose et le Paludisme (FMSTP) qui est un partenariat public privé. L’étude SARA confirme, que les résultats en termes de disponibilité des ressources de base pour la prise en charge du paludisme au niveau des ES de première ligne en Côte d’Ivoire particulièrement dans le secteur public et en zone rurale sont très probants. Cependant la faiblesse des autres composantes du système de santé atténue les efforts quant à la diminution de la morbidité et de la mortalité due au paludisme en Côte d’Ivoire. Cette situation avait été déjà décrite par l’auteur Kerouedan dans un article rapportant l’évaluation de la performance du FMSTP faite en 2008 [13]. Le constat est que 10 ans après la situation n’a pas connue beaucoup d’amélioration. Un autre aspect important dans la prise en charge du paludisme, peu recherché au niveau des pays africains, est le suivi du processus d’utilisation des ressources de base au niveau des ES [14, 15]. Ainsi, avoir des estimations représentatives à l’échelle nationale du processus de prise en charge des cas de paludisme au niveau des ES de première ligne de la Côte d’Ivoire pourraient fournir des éléments essentiels qui permettront d’agir sur le taux élevé de la mortalité due au paludisme. Comme par exemple l’utilisation effective des tests de diagnostic, la proportion de patients ayant le paludisme et recevant un traitement correct, les types de traitements prescrits, le type de recours médicamenteux en cas d’échecs thérapeutiques, la conformité des pratiques aux directives etc. Environ 15% des ES de première ligne ont connu une rupture de stock des CTA dans les 4 semaines précédant l’enquête. Cette situation est malheureusement commune dans les pays de l’Afrique subsaharienne où certaines études ont même donné des taux plus élevés de 25% [16, 17]. Près du quart des ES de première ligne ont rapporté avoir connu des ruptures de stock de TDR dans les 4 semaines ayant précédé l’enquête. Cela reste préoccupant surtout dans les zones rurales des pays en développement où la majorité des ES n’ont pas la capacité de réaliser des gouttes épaisses [18].

Dans la recherche des moyens pour le contrôle et l’élimination du paludisme l’on ne saurait négliger la prévention. Dans notre étude, près du ¾ des ES de première ligne ont disposé de MILDA et de médicaments pour le TPI. Les MILDA sont considérés comme la première stratégie de prévention du paludisme [1, 19]. Cependant, étant donné que le paludisme est une pathologie environnementale et que la majorité des MILDA disponibles dans les ménages sont peu utilisés [20, 21], l’aménagement de l’environnement demeure une question pertinente et un défi au niveau des pays africains. Il a été démontré que les programmes de contrôle du paludisme qui ont mis l’accent sur la gestion de l’environnement ont été très efficaces pour réduire la morbidité et la mortalité [22]. Les leçons tirées de ces programmes réussis peuvent inspirer des approches et des stratégies saines et durables de contrôle du paludisme.

## Conclusion

L’enquête SARA a montré que les ES de première ligne de la Côte d’Ivoire disposaient en 2016 de directives et personnel, de capacité de diagnostic, de médicaments et produits pour une prise en charge efficace des cas de paludisme qui leur étaient adressés avec des disparités selon la zone géographique et l’instance gestionnaire. Pour une meilleure prise en charge du paludisme au niveau des ES de première ligne de la Côte d’Ivoire, il est nécessaire de maintenir la disponibilité des ressources de base existantes avec un renforcement du secteur privé et des zones urbaines. Il est également nécessaire de compléter cette disponibilité par des informations sur le processus de prise en charge du paludisme et de renforcer le système de santé dans son ensemble en plus de la prévention afin d’optimiser la prise en charge du paludisme en Côte d’Ivoire.

### Etat des connaissances actuelles sur le sujet

- En Côte d’Ivoire, le paludisme constitue le premier motif de consultation et d’hospitalisation au niveau des établissements de santé;
- Peu de données sont disponibles sur la capacité des établissements de santé de première ligne à diagnostiquer et traiter les cas de paludisme qui leur sont adressés.

### Contribution de notre étude à la connaissance

- Les établissements de santé de première ligne de la Côte d’Ivoire ont disposé en 2016 de directives et personnel, de capacité de diagnostic, de médicaments et produits pour une prise en charge efficace des cas de paludisme;
- Les ressources de base pour le paludisme étaient plus disponibles dans le secteur public et en zone rurale par rapport au secteur privé et en zone urbaine contrastant avec la faible capacité de ces établissements à fournir des soins de santé généraux aux populations.

## Conflits d’intérêts

Les auteurs ne déclarent aucun conflit d'intérêts.
